# Low-Molecular-Weight Fucoidan Inhibits Thromboinflammation and Ameliorates Deep Vein Thrombosis via Targeting S100A8/A9

**DOI:** 10.3390/md23050180

**Published:** 2025-04-22

**Authors:** Yiting Feng, Weiqing Zhao, Siwen Fang, Jingwen Zhao, Wanshuai Wang, Shaoyun Zhou, Tianyu Wang, Xinke Fang, Xue Chen, Muhammad Awais, Chao Cai, Chuanbin Shen, Ming Liu

**Affiliations:** 1 Key Laboratory of Marine Drugs, School of Medicine and Pharmacy, Ocean University of China, Chinese Ministry of Education, Qingdao 266000, China; fengyiting@stu.ouc.edu.cn (Y.F.); zhaoweiqing@stu.ouc.edu.cn (W.Z.); fangsiwen@stu.ouc.edu.cn (S.F.); zhaojingwen2020@outlook.com (J.Z.); wswang218@163.com (W.W.); zhoushaoyun@stu.ouc.edu.cn (S.Z.); wty1234@stu.ouc.edu.cn (T.W.); fangxinke@stu.ouc.edu.cn (X.F.); chenxue2102@stu.ouc.edu.cn (X.C.); awais119350@gmail.com (M.A.); caic@ouc.edu.cn (C.C.); 2 Laboratory for Marine Drugs and Bioproducts, Qingdao Marine Science and Technology Center, Qingdao 266071, China; 3 Shandong Provincial Key Laboratory of Glycoscience and Glycotechnology, Qingdao 266000, China

**Keywords:** deep vein thrombosis (DVT), thromboinflammation, neutrophil extracellular traps (NETs), polysaccharide, S100A8/A9

## Abstract

Deep vein thrombosis (DVT) is a prevalent life-threatening complication among hospitalized patients. DVT is characterized by the hypercoagulability and thromboinflammation in which platelet activation and neutrophil extracellular trap (NET) formation are critically involved. Studies have shown that S100A8/A9 is significantly elevated in patients with DVT, and is closely associated with platelet activation and NET formation. Fucoidan, the marine polysaccharide derived from Fucus algae, has potential anti-inflammatory and cardioprotective effects. We found low-molecular-weight fucoidan (LMF) bound to S100A8/A9 with an equilibrium dissociation constant (KD) of 2.368 × 10^−8^ M. LMF inhibited S100A8/A9-induced platelet hyperactivity and NET formation in vitro, and ameliorated DVT without significantly perturbing hemostasis in vivo. Our results indicate that the alarmin protein S100A8/A9 is a novel target of LMF. LMF may have therapeutic potential in S100A8/A9-induced thromboinflammation in DVT.

## 1. Introduction

Deep vein thrombosis (DVT), the life-threatening component of venous thromboembolism (VTE), clinically presents as swelling, pain, and ulcer in the lower limbs and is a primary contributor to mortality worldwide [1,2]. DVT is closely associated with thromboinflammation, which can only be partially addressed by conventional antithrombotic treatments [3,4]. Recent studies indicate that, apart from the well-known coagulation activation, platelet activation and the formation of neutrophil extracellular traps (NETs) play essential roles in DVT [5,6]. Activated platelets stimulate endothelial cell activation, facilitating the adhesion of platelets and leukocytes. The activation of platelets and endothelial cells is critical in DVT development, as this process drives the initiation of coagulation [7]. In addition, P-selectin expression on the platelet surface facilitates platelet–neutrophil interactions, promoting NET formation and thrombosis in the inferior vena cava (IVC) [8].

S100A8/A9, also known as calprotectin, is a non-covalently bound heterodimer primarily secreted by activated myeloid cells, possessing proinflammatory and prothrombotic properties [9,10]. Recent research has identified the S100A8/A9-GPIbα axis as a novel mechanism that triggers procoagulant platelet formation [9]. Moreover, as a protein associated with NETs, S100A8/A9 can interact with toll-like receptor-4 (TLR4) on neutrophils, promoting IL-1β secretion and inducing Gasdermin D-dependent platelet pyroptosis through the TLR4-ROS-NLRP3 (NOD-like receptor thermal protein domain associated protein 3)-caspase 1 pathway. This leads to the release of oxidized mitochondrial DNA, contributing to NET formation [11,12]. S100A8/A9 may thus serve as a novel diagnostic marker and therapeutic target for patients with DVT [13].

Fucoidan, a sulfated polysaccharide derived from brown seaweeds and echinoderms, has gained attention for its anti-inflammatory and cardioprotective effects, along with other potential health benefits [14,15]. Our previous study found that low-molecular-weight fucoidan, obtained from fucoidan hydrolysate, exhibited a promising therapeutic potential for thrombus prevention at high blood shear rates [16].

In this study, we found that LMF directly bound to S100A8/A9, with an equilibrium dissociation constant (KD) of 2.368 × 10^−8^ M, and significantly reduced S100A8/A9-mediated platelet activation and NET formation. These findings highlight the potential of LMF for ameliorating DVT by inhibiting S100A8/A9-potentiated prothrombotic and proinflammatory states.

## 2. Results

### 2.1. Structural Characterization and Physicochemical Properties of LMF

**Figure 1:**
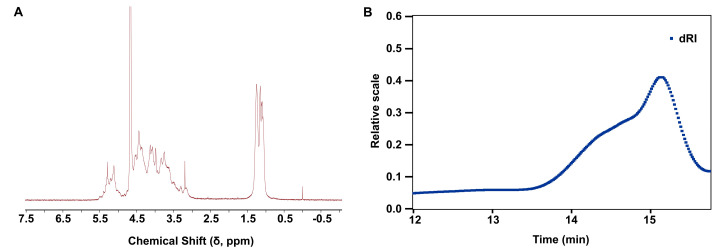
(**A**) The ^1^H-NMR (400 MHz, D_2_O) spectrum of LMF. (**B**) The molecular weight distribution of LMF.

The structural characteristics of LMF are characterized by the analysis of ^1^H-NMR spectrum in Figure 1A. The methyl protons of LMF showed representative chemical shifts at ~1.2 ppm, while the anomeric protons were observed in the downfield region at 5.0–5.5 ppm, indicating the major α-configuration in LMF. The residual signals at 3.5–4.7 ppm were attributed to other protons on the sugar ring, appearing as broad peaks due to heavy overlapping. The molecular weight distribution of LMF, analyzed by multi-angle light scattering (MALS) coupled with size-exclusion chromatography (SEC), revealed a predominant peak centered at approximately 26 kDa (Figure 1B). The dispersity was calculated to be 1.270, as determined directly from the chromatogram using integrated light scattering data.

### 2.2. LMF Inhibited S100A8/A9-Augmented Platelet Aggregation

**Figure 2:**
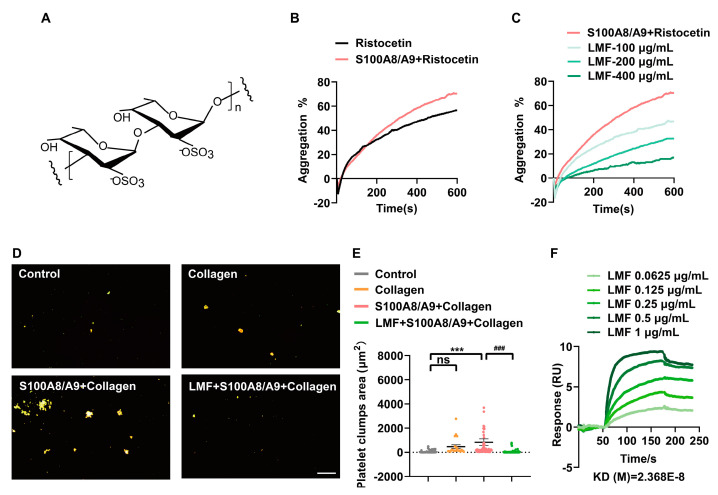
Washed human platelets (2 × 10^8^ cells/mL) were pre-incubated with VWF (2 μg/mL), S100A8/A9 (20 μg/mL), or vehicle (control) for 5 min at 37 °C. Platelet aggregation was carried out in the presence of constant stirring at 1000 rpm/min by an aggregometer. (**A**) Structure of fucoidan from *Fucus vesiculosus*. (**B**) Ristocetin (1 mg/mL)-induced platelet aggregation with S100A8/A9 (20 μg/mL). (**C**) LMF dose-dependent inhibited ristocetin-induced platelet aggregation in the presence of S100A8/A9. (**D**) The effect of LMF (200 μg/mL) on the formation of platelet clumps induced by S100A8/A9 (20 μg/mL) and collagen (2 μg/mL). (**E**) Statistical analysis of the area of platelet clumps. (**F**) Traces from SPR analysis show the interaction between LMF and S100A8/A9 immobilized on Sensor Chip CM5. The values of *p* were determined by one-way ANOVA followed by post hoc Dunnett’s test. ns—not statistically significant (*p* > 0.05); *** *p* < 0.001; ^###^ *p* < 0.001.

S100A8/A9 plays a critical role in the regulation of inflammatory processes and immune response [9]. Recent studies have revealed that S100A8/A9 can induce the formation of procoagulant platelets and thrombosis, suggesting S100A8/A9 is a direct driving force for thromboinflammation [9,12]. To investigate whether fucoidan (Figure 2A) inhibits the S100A8/A9-potentiated platelet activation, we tested the effect of hydrolyzed low-molecular-weight fucoidan (LMF) [16] on platelet aggregation. Consistent with previous findings that S100A8/A9 potentiated ristocetin (an antibiotic that acts by promoting the interaction between the von Willebrand factor (VWF) and the glycoprotein Ib (GPIb) receptor on platelets, leading to platelet clumping)-induced platelet aggregation [9] (Figure 2B), we found that LMF dose-dependently inhibited platelet aggregation triggered by the ristocetin/S100A8/A9 cocktail (Figure 2C). In addition, LMF significantly suppressed the formation of platelet clumps induced by the synergism of S100A8/A9 and collagen (Figure 2D,E). We further proved a direct interaction between LMF and S100A8/A9 (Figure 2F) using surface plasmon resonance (SPR), with a detected association rate constant (*ka*) of 9.723 × 10^4^ Ms^−1^, dissociation rate constant (*kd*) of 2.303 × 10^−3^ s^−1^, and equilibrium dissociation constant (KD) of 2.368 × 10^−8^ M. These findings suggest that LMF may act as an antagonist of S100A8/A9-augmented platelet aggregation through binding to S100A8/A9.

### 2.3. LMF Inhibited S100A8/A9-Induced Platelet Activation and Secretion

**Figure 3:**
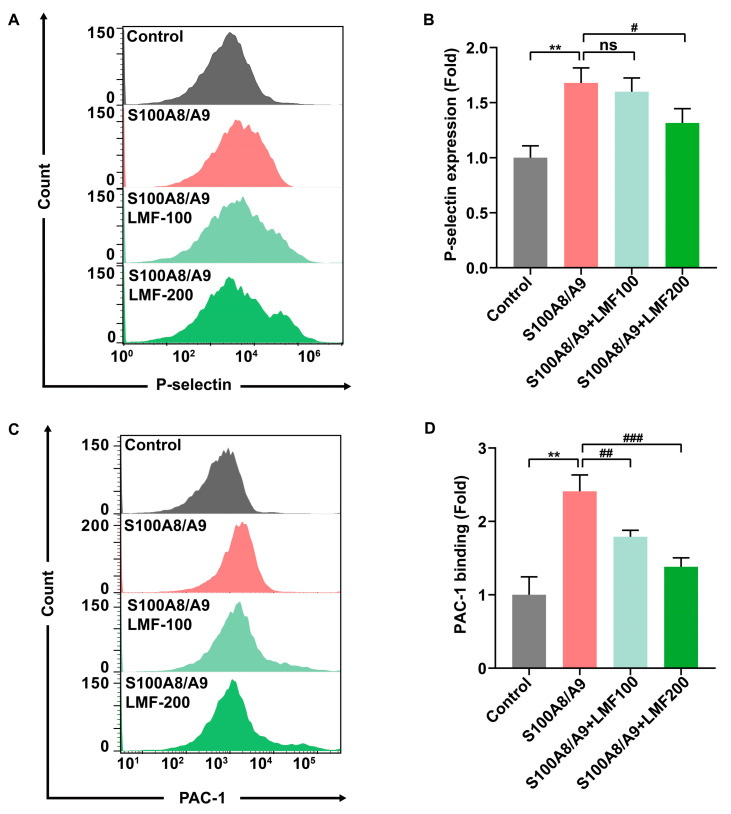
Flow cytometry was employed to assess the effect of LMF on S100A8/A9-induced platelet activation. Isolated human platelets (2 × 10^6^ cells/mL) were incubated with various concentrations of LMF or vehicle (control) for 10 min at 37 °C. (**A**,**B**) The role of LMF (100 μg/mL and 200 μg/mL) in the P-selectin expression on the platelet surface induced by S100A8/A9 (20 μg/mL). (**C**,**D**) The effect of LMF (100 μg/mL and 200 μg/mL) on the PAC-1 binding to platelets induced by S100A8/A9 (20 μg/mL). The values of *p* were determined by one-way ANOVA followed by post hoc Dunnett’s test. ns—not statistically significant (*p* > 0.05); ** *p* < 0.01; ^#^ *p* < 0.05; ^##^ *p* < 0.01; ^###^ *p* < 0.001.

Platelet granule secretion is crucial in the development of DVT [17,18]. Numerous studies have indicated that S100A8/A9 induces α-granule secretion and rapid activation of integrin αIIbβ3 in platelets, thereby promoting platelet activation and platelet-related inflammation [19,20]. To assess the effect of LMF on S100A8/A9-induced platelet activation, flow cytometry analysis was performed. P-selectin, a classical biomarker of platelet secretion, was used as an indicator. LMF significantly inhibited S100A8/A9-induced platelet degranulation, as demonstrated by the reduced expression of P-selectin on the platelet surface (Figure 3A,B). Additionally, LMF decreased S100A8/A9-induced PAC-1 binding to platelets, which is a monoclonal antibody that specifically recognizes the allosterically activated αIIbβ3 (Figure 3C,D). These results indicate that LMF inhibits S100A8/A9-induced platelet activation and secretion.

### 2.4. LMF Inhibited S100A8/A9-Promoted Platelet Adhesion on Immobilized Fibrinogen

**Figure 4:**
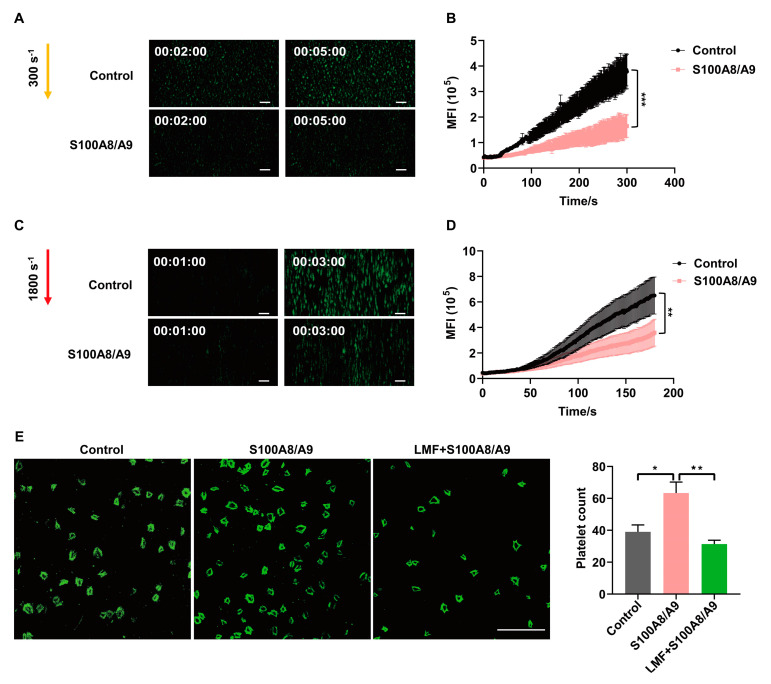
Human whole blood was incubated with S100A8/A9 (20 μg/mL), labeled with DiOC_6_, and then circulated through a collagen-coated μ-slide (ex vivo chamber). Photomicrograph (scale bar: 100 μm) depicting platelet adhesion and thrombus growth under flow conditions of 300 s^−1^ (**A**,**B**) and 1800 s^−1^ (**C**,**D**), respectively. (**E**) The effect of LMF (200 μg/mL) on S100A8/A9-promoted (20 μg/mL) platelet adhesion on immobilized fibrinogen (100 μg/mL). Platelets were incubated at 37 °C for 40 min and stained with FITC-conjugated phalloidin for immunofluorescence microscopy. Scale bar = 10 μm. The values of *p* were determined by one-way ANOVA followed by post hoc Dunnett’s test. * *p* < 0.05; ** *p* < 0.01, *** *p* < 0.001. MFI—mean fluorescence intensity.

Platelet activation and elevated S100A8/A9 levels are associated with DVT [21]. To examine the effect of S100A8/A9 on thrombus formation under flow conditions, we performed perfusion chamber assays using citrate-anticoagulated fluorescently labeled whole blood from healthy volunteers. Surprisingly, we found that S100A8/A9 inhibited thrombus growth under shear rates of both 300 s^−1^ (Figure 4A,B) and 1800 s^−1^ (Figure 4C,D). The interaction between GPIbα and the VWF A1 domain is critical for platelet tethering under conditions of blood shear stress [22]. We found that S100A8/A9 significantly inhibited thrombus formation at different shear stresses (Figure 4A–D). In line with a recent study that showed that S100A8/A9 bound to GPIbα and overlapped with VWF-binding sites [9], this result suggests that S100A8/A9 blocks GPIbα-VWF A1 interaction and may inhibit platelet aggregation under blood shear flow. Therefore, we investigated platelet adhesion and spreading at static conditions in the presence of S100A8/A9 and LMF. LMF significantly inhibited S100A8/A9-promoted adhesions of platelets on the surface of immobilized fibrinogen (Figure 4E), indicating that the activation of integrin αIIbβ3 induced by S100A8/A9 can be significantly inhibited by LMF.

### 2.5. LMF Inhibited S100A8/A9-Induced NET Formation

**Figure 5:**
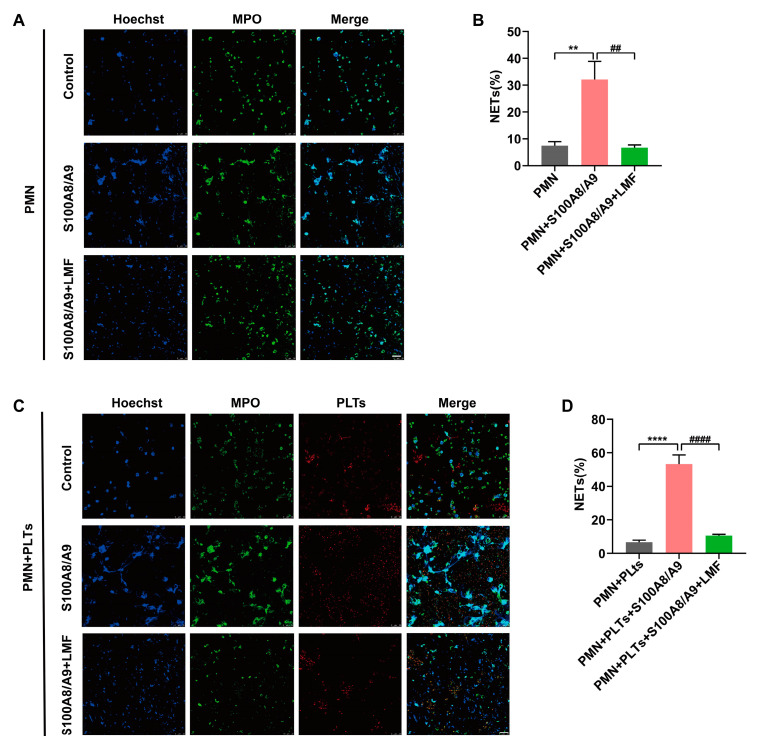
(**A**) Representative confocal images were captured with a 40× water immersion objective (scale bar = 25 μm) in the absence of platelets. (**B**) Quantification of the formation of NETs. (**C**) Representative confocal images were captured in the presence of platelets, and (**D**) quantification of NET formation. Human neutrophils were treated with LMF (200 μg/mL) or vehicle (control) and stimulated with S100A8/A9 (1 μg/mL) in the presence or absence of platelets (PLTs). DNA (blue), NETs (green), and PLTs (red) were stained with Hoechst 33342, anti-MPO antibody, and anti-CD41 antibody, respectively. Results are depicted as mean ± SEM from three independent experiments (n = 3). The values of *p* were determined by one-way ANOVA followed by post hoc Dunnett’s test. ** *p* < 0.01; **** *p* < 0.0001; ^##^ *p* < 0.01; ^####^ *p* < 0.0001. PLTs represents platelets. PMN represents neutrophil.

Neutrophil extracellular traps (NETs) serve as a scaffold for thrombus formation, thereby enhancing thrombus stability and playing a pivotal role in venous thrombosis [23]. To assess the effect of LMF on S100A8/A9-induced NET formation, we performed in vitro NET assays using human neutrophils with or without platelets. Immunofluorescence microscopy of MPO/DNA complexes confirmed that S100A8/A9 significantly promoted NET formation. Meanwhile, S100A8/A9-mediated NET formation was markedly inhibited by LMF in the absence of platelets (Figure 5A,B). Notably, the presence of platelets substantially amplified S100A8/A9-induced NET formation, which was potently suppressed by LMF treatment (Figure 5C,D). These findings demonstrate that LMF directly interacts with S100A8/A9, thereby blocking its capacity to drive NET generation.

### 2.6. LMF Ameliorated DVT Development Without Perturbing Hemostasis

**Figure 6:**
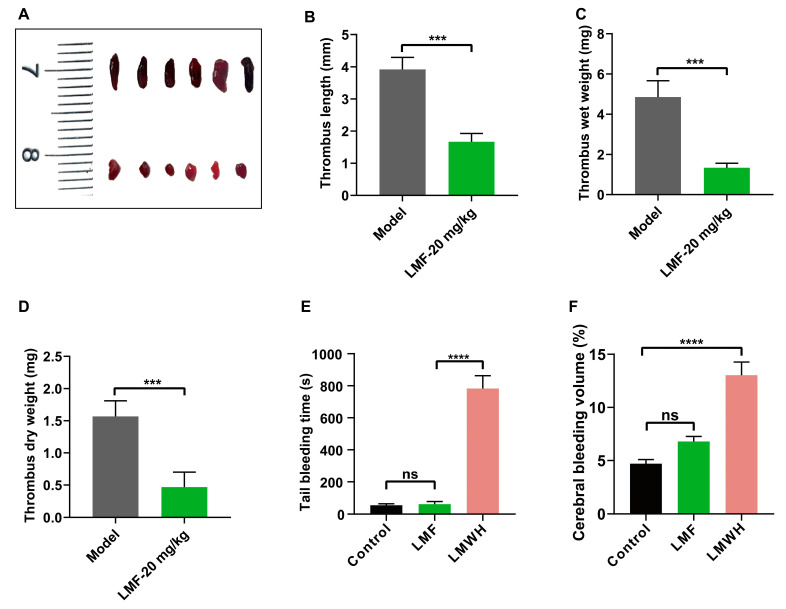
(**A**) Representative images of thrombi from DVT models. Mice (n = 6 per group) received injections of LMF (20 mg/kg) or vehicle (control) subcutaneously. Thrombus was induced by IVC ligation as described in Materials and Methods. After 8 h of surgery, thrombi were excised from IVC for measurements. Statistical analysis of (**B**) thrombus length, (**C**) wet weight, and (**D**) dry weight of the isolated thrombi. (**E**) Tail transection bleeding time following treatment with LMF (20 mg/kg) or low-molecular-weight heparin (LMWH, 4 mg/kg). (**F**) The effect of LMF and LMWH on collagenase-induced cerebral bleeding. The values of *p* were determined by one-way ANOVA followed by post hoc Dunnett’s test. ns—not statistically significant (*p* > 0.05); *** *p* < 0.001; **** *p* < 0.0001.

DVT is a prevalent peripheral vascular disorder that causes mortality in a substantial number of patients each year [24]. Activated platelets facilitated NET formation and enhanced S100A8/A9 levels, which can further contribute to DVT development [25]. We observed that thrombus growth was significantly reduced in LMF-treated mice in an inferior vena cava (IVC) ligation model (Figure 6A–D). Furthermore, tail bleeding assays (Figure 6E) and collagenase-induced cerebral hemorrhage models (Figure 6F) confirmed that LMF did not increase bleeding risk at the same dose as in DVT treatment. These results suggest that LMF alleviates DVT without increasing bleeding risk.

## 3. Discussion

In this study, we demonstrated that the direct interaction between LMF and S100A8/A9 inhibits S100A8/A9-related platelet activation, NET formation, and DVT development. These findings underscore the therapeutic potential of LMF for treating thromboinflammation by disrupting S100A8/A9-mediated downstream signaling transduction on platelets or neutrophils.

Growing evidence suggests that DVT is closely associated with platelet activation, inflammation, and venous stasis at low shear rates (10–100 s^−1^) [26,27,28]. VWF-mediated platelet adhesion to the endothelial cells and leukocyte recruitment through the interaction between platelet GPIbα and VWF play critical roles in the initiation of DVT [29]. The secretion of protein disulfide isomerase (PDI) from endothelial-derived extracellular vesicles (EVs) promotes the exposure of phosphatidylserine (PS) and the release of tissue factor from platelet activation, triggering the extrinsic coagulation pathway and DVT [30,31,32]. Furthermore, VWF-primed platelets recruit neutrophils, while P-selectin and the damage-associated molecular pattern (DAMP) high motility group protein B1 (HMGB1) derived from the activated platelets promote NET formation [33,34]. Recent studies have demonstrated that S100A8/A9 plays a primary role in the development of DVT through inducing platelet activation and NET formation, and S100A8/A9 potentiates VWF-dependent platelet agglutination in the presence of ristocetin and contributes to procoagulant platelet formation and thrombosis [9,12]. In this study, we observed that LMF not only inhibited ristocetin and S100A8/A9-induced platelet aggregation but also prevented or minimized the formation of platelet clumps induced by the synergism of S100A8/A9 and collagen (Figure 2B,D), suggesting a direct blockade effect of LMF on S100A8/A9-related platelet aggregation. Moreover, we indeed identified S100A8/A9 as a novel target of LMF, with an equilibrium dissociation constant (KD) of 2.368 × 10^−8^ M (Figure 2F). This finding may provide an insight into the investigation of the therapeutic potential of LMF in S100A8/A9-related disorders.

S100A8/A9, the alarmin secreted by activated myeloid cells, exhibits antimicrobial, proinflammatory, and prothrombotic properties [9]. The plasma S100A8/A9 level is drastically enhanced in patients with VTE and correlates with thrombosis, which may be a novel diagnostic marker and therapeutic target of VTE [13]. Notably, S100A8/A9 did not directly affect coagulation parameters (including activated partial thromboplastin time and thrombin generation), demonstrating that its prothrombotic effects were mediated through non-coagulation pathways [35]. Studies have shown that S100A8/A9 is the endogenous ligand of TLR4 and GPIbα [9,36]; therefore, it may lead to neutrophil activation and chemotaxis, along with platelet activation [37,38]. Activated platelets directly or indirectly induce NET formation through adhering to neutrophil surfaces and triggering P-selectin/PSGL-1 signal transduction by secreting P-selectin [39,40]. However, we observed that S100A8/A9 promoted NET formation in the absence of platelets (Figure 5A,B), and this process could be augmented in the presence of platelets (Figure 5C,D). Interestingly, LMF dramatically inhibited NET formation irrelevant of platelet presence, suggesting the LMF-S100A8/A9 interaction may block both S100A8/A9-mediated platelet activation and neutrophil activation. Furthermore, LMF effectively reduced thrombus growth in a murine DVT model (Figure 6A–D), which indicates that platelet activation and NET formation are critical for venous thrombus growth. LMF effectively inhibited S100A8/A9-mediated thrombus formation and inflammation by directly binding to S100A8/A9 and probably disrupting its interactions with multiple receptors on platelet and neutrophil. These findings imply that LMF may have a promising therapeutic potential for S100A8/A9-related thromboinflammation, such as in DVT and possibly other conditions.

Fucoidans, the naturally occurring marine polysaccharides derived from brown seaweeds and echinoderms, contain L-fucose subunits with sulfate ester groups (Figure 2A). In general, fucoidans have been recognized as non-anticoagulant sulfated polysaccharides (NASPs) due to their low anticoagulant activity [15,16,41]. Fucoidans have been widely known for their multiple health-beneficial activities such as anti-cancer, anti-infective, immunoregulatory, and cardioprotective effects [16,42,43]. Low-molecular-weight fucoidan (LMF), obtained from fucoidan hydrolysate, prominently inhibited S100A8/A9-mediated platelet activation and NET formation. Furthermore, LMF significantly ameliorated the development of DVT without significant bleeding side effects. Since fucoidan has been widely consumed all over the world as a dietary supplement, clinical investigation is worthwhile to be carried out to confirm the relationship between fucoidan consumption and DVT complications. The limitation of this study was not being able to completely elucidate the structural features of fucoidan and establish the structure–activity relationship between fucoidan and S100A8/A9. This chemical structure–composition–biological activity relationship needs to be investigated in the future.

In conclusion, our study identified S100A8/A9 as a novel target of LMF. LMF significantly inhibited S100A8/A9-related platelet activation and NET formation, thereby ameliorating DVT development. LMF has promising therapeutic potential for reducing S100A8/A9-mediated thrombosis and inflammation.

## 4. Materials and Methods

### 4.1. Analysis of NMR and Physicochemical Properties

The partially hydrolyzed LMF was obtained by dissolving 10 mg of natural fucoidan polysaccharide from *Fucus vesiculosus* (F8190, Sigma, St. Louis, MO, USA) in 1 mL of 0.01 N HCl and heating at 95 °C for 10 min, followed by neutralization with 1 mL of 0.01 N NaOH. The polysaccharide was exchanged with deuterium by lyophilizing it with D_2_O (99.96%, Sigma-Aldrich, Inc., St. Louis, MO, USA) three times. The sample was then dissolved in D_2_O to obtain a ^1^H-NMR spectrum at 25 °C. The ^1^H-NMR spectrum was recorded using an Agilent DD2 400 MHz spectrometer (Agilent, Santa Clara, CA, USA). The molecular weight distribution was measured via multi-angle laser scattering (HPGPC-MALLS) using an Agilent 1260 HPLC system and a DAWN HELEOS-II laser photometer (Wyatt Technology Co., Santa Barbara, CA, USA), equipped with a TSKgel G3000 PWXL (Tokyo, Japan) column (7.8 × 300 mm, 7 μm). The standard product used was Dextran (average mol wt 35,000–45,000, Sigma-Aldrich, USA). This standard was dissolved in the mobile phase (0.1 M NaNO_3_) at a concentration of 5 mg/mL, filtered through a 0.22 μm membrane, and analyzed under chromatographic conditions (TSKgel G3000PWxl column, 35 °C, 0.6 mL/min flow rate, RI detection). The sample was dissolved in the same mobile phase to a final concentration of 10 mg/mL. Chromatographic analysis was performed under isocratic conditions, with a flow rate of 0.6 mL/min and a column temperature maintained at 35 °C.

### 4.2. Animals

BALB/c mice (male, 6–8 weeks old, 20–24 g) were obtained from Jinan Pengyue Experimental Animal Breeding Co., Ltd (Jinan, China). The animals were raised in a controlled laboratory environment (21 ± 2 °C) with a relative humidity of 55–60% and a 12 h light/dark cycle. Mice were grouped and had free access to food and water. All experimental protocols involving mice were approved by the Scientific Ethics Committee of the Academic Committee of Ocean University of China (Approval No. SMP 2023-12-05).

### 4.3. Platelet Isolation and Aggregation Assay

Human platelet-rich plasma (PRP) was prepared from the blood of healthy volunteers who provided informed consent. The blood collection protocol was approved by the Scientific Ethics Committee of the Academic Committee of Ocean University of China (Approval No. HM-2023R81). Platelets were isolated from PRP through washing and resuspending in Tyrode’s buffer as previously described [44,45]. Aggregation assays were performed as per prior methods [46,47,48]. In short, washed platelets were incubated with different concentrations of LMF or vehicle. After 5 min at 37 °C, platelet aggregation was initiated by adding recombinant human S100A8/A9 heterodimer protein (CT002-H0822B, Sinobiological, Beijing, China) and monitored by a Techlink aggregometer (LBY-NJ4, Beijing, China).

### 4.4. Flow Cytometry

Integrin αIIbβ3 activation and P-selectin expression were evaluated using FITC-conjugated PAC-1 (1: 100, MA528564, Thermofisher, Waltham, MA, USA) and PE-conjugated P-selectin antibodies (1:800, 12-0626-82, Invitrogen, Carlsbad, CA, USA), with slight modifications from previous methods [16,44]. Platelet suspensions (2 × 10^6^ cells/mL) were incubated with LMF or S100A8/A9 in the dark at 37 °C for 30 min. The levels of PAC-1 binding and P-selectin expression were assessed using a flow cytometer (MoFlo™ XDP, Beckman Coulter, Brea, CA, USA,) and analyzed with FlowJo™ v10 software.

### 4.5. Surface Plasmon Resonance (SPR) Analysis

SPR analysis was performed as previously described with minor modifications [44,49]. Briefly, recombinant human S100A8/A9 heterodimer protein was immobilized on an activated Sensor Chip CM5 (BR100530, Cytiva, Marlborough, MA, USA) by amine coupling, and LMF in a PBSP running buffer was injected as the mobile ligand at a flow rate of 10 μL/min, with real-time binding signals recorded using Biacore™ T200 Evaluation software 3.2 (GE Healthcare, Milwaukee, WI, USA).

### 4.6. Ex Vivo Perfusion Chamber

To investigate the impact of S100A8/A9 on thrombus formation under different shear conditions, an ex vivo perfusion chamber system was established as previously described [16]. The μ-Slide (μ-Slide VI 0.1, ibidi, Bavaria, Germany) was coated with 50 μg/mL of collagen (AG005K, HYPHEN, IDF, France) and incubated overnight at 4 °C. Sodium citrate-anticoagulated whole blood samples from healthy volunteers were labeled with 3,3′-dihexyloxacarbocyanine iodide (1 μM, DiOC_6_, Sigma) and subsequently preincubated with S100A8/A9 for 5 min at room temperature. The blood was circulated over the collagen-coated surface using a syringe pump (TYD01-01, Lead Fluid, Baoding, China) at shear rates of 300 s^−1^ and 1800 s^−1^. Thrombus formation was monitored in real-time using a fluorescent microscope (Novel Optics, Ningbo, China, NIB 900 inverted) equipped with a 10× objective, and quantitative dynamics of platelet fluorescence intensity were analyzed with SlideBook software 5.5 (Intelligent Imaging Innovations, Denver, CO, USA).

### 4.7. Platelet Adhesion and Confocal Microscopy

The impact of LMF on S100A8/A9-mediated platelet adhesion was determined according to the method we previously described [44,48]. Shortly, washed platelets suspended in Tyrode’s buffer were incubated with either LMF or S100A8/A9 at 37 °C and then added to coverslips (20 mm) coated with 100 μg/mL of fibrinogen (S12023, Yuanye, Shanghai, China). Platelets were then fixed with 4% paraformaldehyde, permeabilized with 0.3% Triton X-100 for 10 min, and blocked with 2% BSA in PBS for 60 min. Samples were then stained with FITC-labeled phalloidin overnight in the dark at 4 °C and examined using a confocal microscope (DMi8, Leica, Wetzlar, Germany). The platelet spreading area was quantified with ImageJ software (version 1.35h).

### 4.8. Isolation of Neutrophils and NET Formation Assay (In Vitro)

Neutrophils were isolated from healthy donors using the Human Peripheral Blood Neutrophil Isolation Kit (P9040, Solarbio, Beijing, China) with modifications [8,50]. Initially, sodium citrate-anticoagulated blood was separated using a gradient separator and centrifuged (1000 *g*, 30 min) at room temperature. The neutrophil layer was carefully collected, washed with PBS, purified using a red cell lysis buffer, and then resuspended in a pre-warmed RPMI 1640 medium with a 1% penicillin/streptomycin mixture. Neutrophils (1 × 10^7^ cells/mL) were placed on polylysine-coated slides at 37 °C for 30 min, with or without platelets treated with LMF or S100A8/A9. Immunofluorescence staining was performed using anti-human CD41a (133903, Biolegend, San Diego, CA, USA), MPO monoclonal antibody (11-1299-42, Invitrogen), and Hoechst 33342 (HY-15559, MCE, Monmouth Junction, NJ, USA), and NET formation was observed by confocal microscopy.

### 4.9. Murine Model of Deep Vein Thrombosis

A deep-vein thrombosis (DVT) model was used to examine the effect of LMF on venous thrombosis, as previously described [51]. Mice were divided into two groups (n = 6, subcutaneous injection): a control group (vehicle only) and an LMF-treated group. Animals received treatment before inferior vena cava (IVC) ligation and were anesthetized with an isoflurane/oxygen mixture (1.5–2%, 1 mL/min) in the induction chamber, then transferred to a nose cone for continuous anesthesia (0.6 mL/min) using an anesthesia apparatus (R500IE, RWD Life Sciences, Shenzhen, China). A median laparotomy was performed to isolate the IVC, followed by ligation below the left renal vein using a 7.0 polypropylene filament (Premilene^®^, Braun, Melsungen, Germany) to completely obstruct the blood flow. Visible side branches were manipulated or ligated to ensure complete occlusion. Immediately after intervention, the peritoneum and skin were sutured with a monofilament 6.0 silk suture (Ethicon, Somerville, MA, USA). Mice showing bleeding or IVC injury during the procedure were excluded. After 8 h, the mice were euthanized, IVCs were excised below the renal veins and developed thrombi were measured.

### 4.10. Tail Bleeding

Male BALB/c mice (7–8 weeks old) were injected intravenously with either LMF (20 mg/kg) or LMWH (4 mg/kg). After 10 min, a 5 mm segment of the tail tip was transected and immersed in normal saline at 37 °C. Bleeding time was recorded until cessation for 15 min.

### 4.11. Collagenase-Induced Cerebral Bleeding

Male BALB/c mice (7–8 weeks old) were anesthetized with isoflurane delivered by an anesthesia respirator after intravenous administration of LMF (20 mg/kg) or LMWH (4 mg/kg). Following aseptic preparation of the surgical site, a midline scalp incision was made to expose the skull, and a 1 mm burr hole was drilled 2.0 mm to the right of the fontanelle and 0.3 mm anterior to the bregma. A micro-syringe was inserted vertically 3.5 mm below the dura mater to slowly and steadily infuse 0.2 units of collagenase IV in saline. The skin was sutured, and the wound was disinfected with iodine. After 24 h, the mice were euthanized, the brains were harvested and sectioned into 2 mm thick slices, and the hemorrhage area was measured using ImageJ.

### 4.12. Statistical Analysis

Statistical analysis was performed using one-way ANOVA followed by post hoc Dunnett’s test in GraphPad Prism 9.0.0.121 (San Diego, CA, USA). Results were reported as means ± SEM (standard error of the mean). *p*-values of 0.05 or less were considered statistically significant.

## Data Availability

The original data presented in the study are included in the article; further inquiries can be directed to the corresponding authors.
